# Whipple’s endocarditis diagnosed non-invasively with plasma microbial cell-free DNA sequencing

**DOI:** 10.1016/j.idcr.2025.e02308

**Published:** 2025-07-03

**Authors:** Najy Issa, Zaid Naseer, Andrew Mangano, Norman Bernstein

**Affiliations:** a Department of Internal Medicine, Mary Washington Healthcare, Fredericksburg, VA 22401, USA; b Department of Infectious Diseases, Mary Washington Healthcare, Fredericksburg, VA 22401, USA

**Keywords:** Whipple's Endocarditis, Microbial Cell-Free DNA, NGS, Tropheryma Whipplei, Culture-negative Endocarditits, Bioprosthetic Aortic Valve

## Abstract

*Tropheryma whipplei* bioprosthetic aortic valve endocarditis was diagnosed in a 55-year-old patient who presented with neurologic signs. Next-generation sequencing (NGS) of microbial cell-free DNA (cfDNA) detected a profound level of the organism in a plasma sample. This entity often comes to diagnosis only after histopathology and molecular diagnostic techniques are applied to infected heart valves in cases of culture-negative endocarditis. After 12 months of therapy, this patient has avoided valve replacement.

## Introduction

Whipple's disease is a rare, chronic, and systemic infection caused by the bacterium *Tropheryma whipplei*. The organism is a gram-positive bacillus that primarily affects the small intestine, leading to malabsorption and weight loss. It can also involve other organs, including the heart, where it manifests as Whipple’s endocarditis. Diagnosing this condition has traditionally been challenging due to its nonspecific symptoms and the limitations of conventional diagnostic methods [1]. However, recent availability of next-generation sequencing (NGS) of microbial cell-free DNA (cfDNA) in plasma has provided an additional tool to determine the microbial etiology of endocarditis when routine blood cultures are negative [2]. It provided the sole microbiologic result in this case to guide specific antibiotic therapy.

## Case report

A 55-year-old man with a bovine aortic valve placed 4 years earlier for atherosclerotic aortic stenosis verified by pathology exam presented to the emergency department (ED) with a 6-day history of diplopia and slurred speech. He also reported intermittent arthralgias in the hands and both wrists for several months. Non-contrast MRI of the brain revealed acute infarctions in the left midbrain and cerebellum. While under evaluation in the first 48 h, the neurologic symptoms completely resolved.

In the prior 4 years after cardiac surgery, the patient experienced a bout of atrial fibrillation treated with anticoagulation, episodes of syncope attributed to an immunization and fecal impaction, a stroke, and one seizure. Anticonvulsant treatment was declined and there were no additional seizures. Thirty years earlier, the patient had undergone treatment for stage 3 Hodgkin’s lymphoma with chemotherapy and radiotherapy to the chest.

**Fig. 1:**
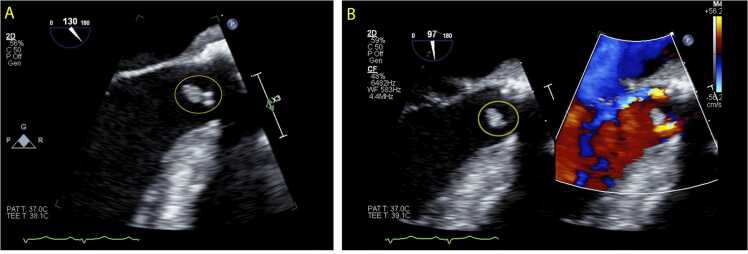
Transesophageal echocardiogram long axis views (A,B) show a 1.3 cm mobile vegetation on the aortic valve, prior to the initiation of antibiotics.

In the Observation Unit, vital signs showed a tachycardic heart rate of 130, but no temperature elevation. The remainder of the physical exam was normal. Upon Neurology consultation and review of the history of previous neurologic events and imaging, Transesophageal Echocardiogram (TEE) was recommended to evaluate the bioprosthetic valve for signs of endocarditis. TEE confirmed a 1.3 cm vegetation on the aortic valve (Figs. 1A, 1B). Laboratory findings showed elevated white blood cell count of 17.07 k/µl, lactic acid of 2.50 mmol/L, CRP of 35 mg/L, and ESR of 58 mm/h.

A comprehensive infectious, autoimmune, and metabolic workup was performed. Infectious testing ruled out tuberculosis, HIV, syphilis, hepatitis B and C, *Bartonella*, and *Coxiella burnetii*. Thyroid function tests showed no evidence of thyroid dysfunction. Blood and cerebrospinal fluid (CSF) cultures were negative. Lumbar puncture revealed clear CSF with elevation of protein 81 mg/dL (17–65 mg/dL), normal glucose, CSF WBC count of 0 cells/cu mm and red blood cell count of 11 cells/cu mm. An ophthalmologic examination showed no signs of uveitis or retinitis. Autoimmune evaluation for antinuclear antibody (ANA), rheumatoid factor (RF), anti-cyclic citrullinated peptide (anti-CCP), and antineutrophil cytoplasmic antibodies (ANCA) were all negative. *Tropheryma whipplei* DNA PCR on CSF was undetectable.

A plasma sample for metagenomic testing was submitted, and the patient was empirically started on intravenous Vancomycin and Ceftriaxone. The patient soon complained of intolerance to Vancomycin, which was discontinued. Ceftriaxone 2 g intravenously daily was continued.

Plasma metagenomic cfDNA sequencing was performed at Karius, Inc., Redwood City, CA, using proprietary next-generation sequencing technology [3], [4]. Results were reported within 3 days and revealed *Tropheryma whipplei* 16,219 DNA molecules per microliter (MPM) (reference range < 10 MPM). Follow-up metagenomic testing as an outpatient could not be performed because of the prohibited cost.

**Fig. 2:**
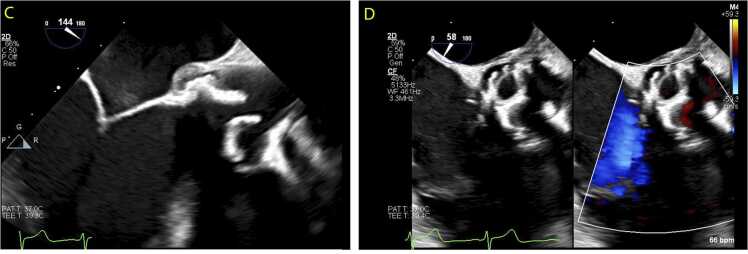
Transesophageal echocardiogram long axis (C) and short axis (D) views showing resolution of previously noted vegetation after 6 weeks of antibiotics.

*T. whipplei* DNA detection by PCR on plasma was drawn after 2 weeks of Ceftriaxone therapy and PCR was indeterminate. At that time, the patient reported resolution of arthralgias. Follow-up TEE at 6 weeks showed resolution of the vegetation (Figs. 2C, 2D). He was then transitioned to double-strength trimethoprim-sulfamethoxazole (TMP-SMX) and completed 12 months of treatment.

For post-treatment monitoring, plasma *T. whipplei* PCR and echocardiography are planned at 3-month intervals for the first year after initiation of therapy. Testing will be extended based on clinical findings. At 12 months post-treatment, he has shown a decreased left ventricular ejection fraction but no sign of valvular relapse.

## Discussion

*T. whipplei* infective endocarditis (TWIE), a rare manifestation of Whipple's disease, involves infection of the heart valves. Patients may present with neurologic signs, arthralgias, and heart failure. TWIE most frequently presents as a blood culture-negative endocarditis on a native valve, with prosthetic valve endocarditis rarely reported [1].

While gastrointestinal manifestations, weight loss, and diarrhea are often seen in classical Whipple’s disease, our patient denied any such history. There was no evidence of lymphadenopathy or unexplained constitutional symptoms in prior years, which supports this was more likely a localized cardiac infection. Although the neurologic presentation was ultimately attributed to embolic stroke, the preceding joint symptoms were likely due to localized *T. whipplei* infection of the prosthetic aortic valve—an often overlooked but frequently reported feature in such cases [1].

This patient had no known ongoing immunosuppressive condition, but his remote history of chemotherapy and radiotherapy for Hodgkin’s lymphoma likely contributed to long-term immune dysregulation. He was not on immunosuppressive medications and had no evidence of HIV or autoimmune disease.

Traditionally, the standard approach for evaluation of Whipple’s endocarditis has been the histological examination of cardiac valve tissue and molecular diagnosis (e.g., 16S rRNA gene PCR/sequencing, *T. whipplei* PCR) of excised cardiac valves [1]. TWIE typically comes to diagnosis late in the patient’s course requiring open-heart surgery. Our patient would find it challenging to undergo open heart surgery given his prior chest radiotherapy and valve surgery.

NGS technology enables sequencing of microbial cfDNA circulating in the bloodstream. This approach allows detection of a wide range of pathogens, including bacteria, viruses, fungi, and parasites, directly from plasma samples without invasive procedures. A prior case of TWIE was identified using NGS showing 766 MPM—far less than the 16,219 MPM seen in our case [5]. The clinical course of that earlier case was not reported. The International Society for Cardiovascular Infectious Diseases Criteria for Infective Endocarditis Working Group believes that a positive result for *Coxiella burnetii*, *Bartonella* species, or *T. whipplei* from a metagenomic sequencing platform should constitute a Major Criterion in the updated Modified Duke Criteria for infective endocarditis [6].

*T. whipplei* DNA PCR test used here typically provides a detected or undetected qualitative result based on a DNA probe match to a primer pair TwrpoB.F and TwrpoB.R. A DNA probe to only one and not both, results in an indeterminate reading. There is limited clinical data to evaluate how well this test performs in different disease states. There is no specific medical recommendation to support its use to monitor for relapse of infection here. Follow-up assessments with appropriate history, physical examination, standard laboratory and imaging studies should be obtained. *T. whipplei* is notoriously difficult to isolate using standard blood or tissue cultures. As a result, molecular diagnostics play a key role in detection. While direct serum PCR has limited sensitivity, its positive predictive value remains high [1], [7], [8]. Further investigation should be pursued to explore the role of routine direct serum PCR monitoring in Whipple’s endocarditis.

Unfortunately, relapse of TWIE remains a significant concern, largely due to the organism’s intracellular persistence and the difficulty of achieving complete eradication, particularly in valvular tissue. Although exact relapse rates are not well defined due to limited data, the potential for asymptomatic carriage further complicates management [7].

There is no established standard treatment for TWIE*,* particularly in the context of prosthetic valve involvement. In this case, the patient received six weeks of intravenous ceftriaxone followed by oral DS TMP-SMX for 12 months. This regimen was chosen based on the need for agents with prolonged intracellular activity and adequate cardiac tissue penetration. The antibiotic duration was intended to minimize the risk of relapse, given the organism’s persistence within macrophages and the added challenge of sterilizing prosthetic material, where antibiotic penetration is often limited [7], [4]. Furthermore, our choice of antibiotic duration aligns with previously published data, which reported no relapses with a 12-month course of ceftriaxone and TMP-SMX [8], [9].

Virtually all patients described in the literature with this condition have ultimately required surgical valve replacement. This may also be the eventual outcome for our patient, who has a persistently reduced ejection fraction and residual aortic insufficiency. However, an initial attempt to avoid open-heart surgery was warranted given his history of prior mediastinal radiation and the associated elevated surgical risk.

## Conclusion

The use of NGS for detecting microbial cfDNA in plasma for *Tropheryma* proved to have several advantages over traditional diagnostic methods for this patient:

• Non-invasiveness: NGS required only a blood sample and facilitated early diagnosis. This allowed for targeted medical therapy while potentially delaying the need for surgical intervention in a high-risk patient with prior cardiac surgery and chest radiotherapy.
• Rapid Turnaround: NGS provided results within a few days, compared to the extended time required for traditional cultures and histological examinations. Our patient, initially treated empirically with Vancomycin and Ceftriaxone, complained of intolerance to one of his antibiotics. We discontinued Vancomycin but were fortunate to have a quick turnaround result with the metagenomic test to confirm an appropriate plan of treatment with Ceftriaxone. Later, we transitioned to an extended course of DS TMP-SMX with the goal of reducing the risk of relapse and maintaining disease control.
• High Sensitivity: NGS can detect low levels of pathogen DNA, which may be useful for diagnosing cases of culture-negative endocarditis in the community hospital setting.

## Author Contributions

**Najy Issa, MD** – Primary author; case analysis, literature review, manuscript drafting and revisions. **Zaid Naseer, MD** – case analysis, literature review, and manuscript editing. **Andrew Mangano, DO** – case analysis, manuscript drafting and revisions. **Norman Bernstein, MD** - Contributed to clinical care, case analysis, literature review, manuscript drafting and revisions.

## CRediT authorship contribution statement

**Zaid Naseer:** Writing – review & editing, Writing – original draft, Formal analysis. **Najy Issa:** Writing – review & editing, Writing – original draft, Investigation, Formal analysis. **Andrew Mangano:** Writing – review & editing. **Norman Bernstein:** Writing – review & editing, Writing – original draft, Supervision, Formal analysis.

## Consent

Signed consent obtained.

## Ethical approval

Yes.

## Ethical considerations

This case report does not contain any identifiable patient information. Written informed consent was obtained from the patient for publication.

## Author’s statement

We confirm that all authors have made substantial contributions to the conception, design, drafting, and critical revision of this manuscript and have approved the final version for submission. A competing interests disclosure has already been submitted separately.

## Funding

No external funding was received for the preparation of this manuscript.

## Declaration of Competing Interest

The authors declare that they have no known competing financial interests or personal relationships that could have appeared to influence the work reported in this paper.
